# A randomized controlled trial evaluating the effects of motivational interviewing in new hearing aid users (MI-HAT): study protocol for a randomized controlled trial

**DOI:** 10.1186/s13063-023-07352-7

**Published:** 2023-05-22

**Authors:** Alice Q. Liu, Printha Wijesinghe, Melissa Lee, Carol Lau, Jane Sun, Desmond A Nunez

**Affiliations:** 1 Division of Otolaryngology – Head & Neck Surgery, University of British Columbia, Vancouver Coastal Health, 2775 Laurel St, 4th floor, BC V5Z 1M9 Vancouver, Canada; 2 Faculty of Medicine, University of British Columbia, Vancouver, BC Canada; 3 Sound idEARS Hearing Clinic, Vancouver, BC Canada; 4 BC Mental Health and Substance Use Services, BC Vancouver, Canada

**Keywords:** Motivational interviewing, Hearing aid, Hearing loss, Randomized controlled trial, Study protocol

## Abstract

**Background:**

Hearing loss is the third leading global cause of disability and is associated with poorer quality of life. Hearing aids are often recommended for hearing loss; however, hearing aid uptake and use rates are perpetually low. Motivational interviewing (MI) is a patient-centered counseling aimed at addressing the desire in the patient to change their behavior. The aim of this study is to investigate the impact of one-on-one MI sessions on hearing aid use among new adult users.

**Methods:**

A multi-center, prospective, randomized patient-blind controlled trial with a pre- and post-tests design. New hearing aid users ≥ 18 years of age will be recruited from Vancouver, Canada. They will be randomly assigned to a treatment or control group. The treatment group will attend a one-on-one MI session hosted by a practicing MI therapist in addition to standard in-person audiological care. The control group will receive standard in-person audiological care. Data is collected at baseline and at 1, 3, 6, and 12 months’ follow-ups. The primary outcomes are data-logged hearing aid use hours and patient-reported outcomes as measured by the International Outcome Inventory for Hearing Aids questionnaire. Associations between intervention and hearing aid use hours and self-reported outcome measures will be assessed.

**Discussion:**

This trial is designed to evaluate the efficacy of one-on-one MI in improving hearing aid use in new adult users in the short and long terms. Results will contribute to the evidence on whether MI counseling has an effect on hearing aid use and may guide future clinical practices.

**Trial registration:**

ClinicalTrials.gov NCT04673565. Registered on 17 December 2020.

**Supplementary Information:**

The online version contains supplementary material available at 10.1186/s13063-023-07352-7.

## Administrative information

Note: the numbers in curly brackets in this protocol refer to SPIRIT checklist item numbers. The order of the items has been modified to group similar items (see http://www.equator-network.org/reporting-guidelines/spirit-2013-statement-defining-standard-protocol-items-for-clinical-trials/).

**Table:** 

Title {1}	A randomized controlled trial evaluating the effects of motivational interviewing in hearing aid users (MI-HAT): study protocol
Trial registration {2a and 2b}.	ClinicalTrials.gov Review Board NCT04673565
Protocol version {3}	2022-08-17 Version 9.0
Funding {4}	None.
Author details {5a}	Division of Otolaryngology Head & Neck Surgery, University of British Columbia. Vancouver, BC, Canada.
Name and contact information for the trial sponsor {5b}	University of British Colombia Gordon and Leslie Diamond Health Care Centre 4^th^. Floor-2775 Laurel Street DHCC, Vancouver General Hospital Vancouver, BC V5Z 1M9 Phone: (+1) 604.875.4664 desmond.nunez@ubc.ca
Role of sponsor {5c}	The sponsor played no part in study design; collection, management, analysis and interpretation of data; writing of the report; and the decision to submit the report for publication.

## Introduction

### Background and rationale {6a}

#### Hearing loss

Sensorineural hearing loss can be defined as reduced hearing ability secondary to dysfunction of the inner ear or auditory nerve. Presbycusis, or age-related hearing loss, is the most common form of sensorineural hearing impairment.

Hearing impairment is associated with anxiety, depression, safety concerns, lack of mobility, and reduced employment opportunities and income [1]. The 2019 Global Burden of Disease Report estimates that 1.57 billion people worldwide suffer from hearing loss and that hearing loss is the third leading cause of human disability [2].

#### Hearing aids

Many studies demonstrate that hearing aid (HA) use increases the user’s ability to detect, differentiate, and locate sound and improve speech recognition and health-related quality of life (HRQoL). A review by Chisolm et al. [3] assessed the effect of amplification on HRQoL and concluded that HAs had a medium-to-large effect. Other reviews on the effects of HAs on quality of life have reached similar conclusions [4, 5].

Despite these findings, HA uptake and use are generally poor. A UK National Health Service survey indicated that approximately 40% of new patients use their HAs for less than 4 h a day [6]. Similar conclusions have been reported in other comparable countries [7, 8]. Reasons for this lack of use include disappointment with HA effectiveness, perceived lack of need for a HA, affordability or cost concerns, and the social stigma [9–11].

#### Person-centered counseling

Person-centered counseling has been popularized in audiology literature in the past decade. Person-centered counseling, initially developed by Carl Rogers, facilitates the discovery of the client’s own intrinsic tendency toward healing and growth [12]. Laplante-Lévesque et al. found that the therapeutic relationship between the audiologist and the patient is an important determinant of HA use [12, 13]. Motivational interviewing is one potential intervention that may be utilized to increase HA use.

#### Motivational interviewing

Motivational interviewing (MI) is a patient-centered counseling technique that describes readiness to change as a dynamic rather than a static state [14]. MI is aimed at eliciting behavioral change by exploring and resolving a patient’s ambivalence. MI has been successfully used to increase adherence to long-term treatment and promote behavioral change in other medical fields [15–17].

Several studies have investigated the impact of MI on HA use [6, 18, 19]. However, these studies have had significant limitations. Aazh et al. [6] and Ferguson et al. [19] studies were only pilot studies and had limited power, while Solheim et al. [18] did not use a control group. Furthermore, none of the above studies considered the long-term outcomes of MI and whether these positive results would persist. A recent systematic review found no robust evidence that MI significantly improved HA use or user-reported outcomes, and concluded that future research is warranted [20].

#### Justification

Hearing loss is the third leading cause of disability globally and HA use can improve a client’s hearing ability and quality of life. MI is a technique shown to be effective in addressing other chronic conditions and so far, definitive evidence of its efficacy or otherwise in the hearing-impaired population is lacking. The investigative team of this study includes an experienced clinical trialist, otolaryngologist, motivational therapist, and dispensing audiologists. These experts designed a randomized controlled trial study protocol to determine the efficacy of the intervention, one-on-one MI, in improving adherence to HA use in new clients in the short and long terms.

### Objectives {7}

The primary objective is to determine the efficacy of one-on-one MI in increasing adherence to HA use in new adult clients in the short and long terms. The secondary objective is to determine if there are adverse effects associated with one-on-one MI.

### Hypothesis

Standard audiological care with one-on-one MI does not alter HA use compared to standard audiological care alone in new adult HA clients.

### Trial design {8}

This study will be a multi-center, prospective, randomized patient-blind controlled trial and employ a between-subject, pre- and post-tests design with a treatment and control group. It has been designed to be a superiority trial assessing motivational interviewing compared to standard audiological care. The SPIRIT (Standard Protocol Items: Recommendations for Interventional Trials) reporting guidelines were used to develop the methodology.

## Methods: participants, interventions, and outcomes

### Study setting {9}

Study participants will be recruited at audiology clinics located within the greater Vancouver region, British Columbia (BC), Canada. Audiology clinics will comply with WorkSafeBC and the Provincial Health Officer’s COVID-19 orders as related to safety plans. Audiologists will screen potential participants during the normal course of their work.

### Eligibility criteria {10}

Study eligibility criteria include new HA users who are **≥** 18 years old, with a Pure Tone Audiometric (PTA) hearing threshold above 25 dB in the worse ear at frequencies averaged across either 0.5, 1, 2, and 3 kHz or 4, 6, and 8 kHz (Table 1).

**Table 1:** Inclusion and exclusion criteria

**Criteria**	**Description**
Inclusion	• 18 years and older • New hearing aid user • Unilateral or bilateral PTA hearing threshold above 25 dB in the worse ear across either 0.5, 1, 2, and 3 kHz, or 4, 6, and 8 kHz
Exclusion	• History of hearing aid use • Do not understand the English language • Unable to complete the online questionnaires in English language • Have inconsistent pure-tone audiometric readings • Have medical constraints that prohibit them from wearing hearing aids

Participants who have a history of HA use, do not understand English, do not respond reliably to audiometry, or have concurrent medical contradictions to HA use will be excluded.

Participants will be invited by the audiologists to undergo baseline assessment after screening.

### Who will take informed consent? {26a}

Written informed consent for study participation will be obtained by trained audiology clinic assistants. Completed consent forms will be forwarded to the study coordinator for secured documentation.

### Additional consent provisions for collection and use of participant data and biological specimens {26b}

No additional consent provisions for collection and use of participant data and biological specimens will be required.

### Interventions

#### Explanation for the choice of comparators {6b}

Participants in the control group will receive standard care as typically delivered by audiologists at in-person clinic visits with no additional treatment. Above comparator was selected to allow the effect of one-on-one MI on adherence to HA use to be compared with the current gold standard treatment of standard care. Audiologists will make treatment plans for new HA clients based on their personal experience, the evidence available from published studies, and expert opinion. There was no attempt to limit audiologists who deliver care in the comparator arm to ensure that the study outcomes can be generalized to the practice of non-study audiologists.

#### Intervention description {11a}

Patients allocated to the intervention group will attend a 30-min maximum one-on-one MI session hosted by a practicing MI therapist via Zoom at 1 month after the initial visit. Participants will also receive standard audiological care delivered at in-person clinic visits.

All control and intervention participants will be asked to attend 4 follow-up visits after the initial baseline assessment at 1, 3, 6, and 12 months. At follow-up visits, all participants will complete online International Outcome Inventory for Hearing Aids (IOI-HA) questionnaires and the study investigators will access each participant’s HA data-logging feature to determine the mean number of hours HAs were activated. Audiologist(s) will record the daily average hours of use based on the number of days since the last reading. All data will be entered using a secure online survey tool.

#### Criteria for discontinuing or modifying allocated interventions {11b}

The intervention and control groups are expected to have a low risk of adverse events. Interim visits not previously scheduled may be required for other reasons such as HA malfunctions. All events of accidental injury or participant withdrawal of consent during the trial will be recorded.

#### Strategies to improve adherence to interventions {11c}

At each study visit, adherence to the study intervention will be assessed in both the intervention and control groups. Strategies to improve adherence include:

- Individual assessment and instruction by study investigators and those involved.
- Participants will be contacted directly by telephone by study investigators if there are missed visits to encourage continued participation and to evaluate/assess barriers that prohibit participation.
- Telehealth follow-up visits with participating audiologists will be offered to those who may have limited access or who prefer telehealth.

#### Relevant concomitant care permitted or prohibited during the trial {11d}

There are no prohibitions on participants seeking or receiving unplanned care for HA malfunction or ear complaints during the course of the trial. All such visits and their purpose will be recorded.

#### Provisions for post-trial care {30}

There are no planned provisions for post-trial care other than standard care for HA clients which includes follow-up visits.

### Outcomes {12}

#### Primary outcomes

The primary outcome is the inter-group difference in average hours of HA use at 1, 3, 6, and 12 months post-intervention aimed at assessing the early and long-term effect of the intervention. This is a quantitative measure recorded by the HAs data-logging feature.

The secondary outcomes are differences in inter-group average participant-completed questionnaire (IOI-HA) [21] scores at 1, 3, 6, and 12 months post-intervention and reported complications. The IOI-HA is an established rigorous patient reported measure for HA effectiveness [22, 23].

### Participant timeline {13}

**Fig. 1:**
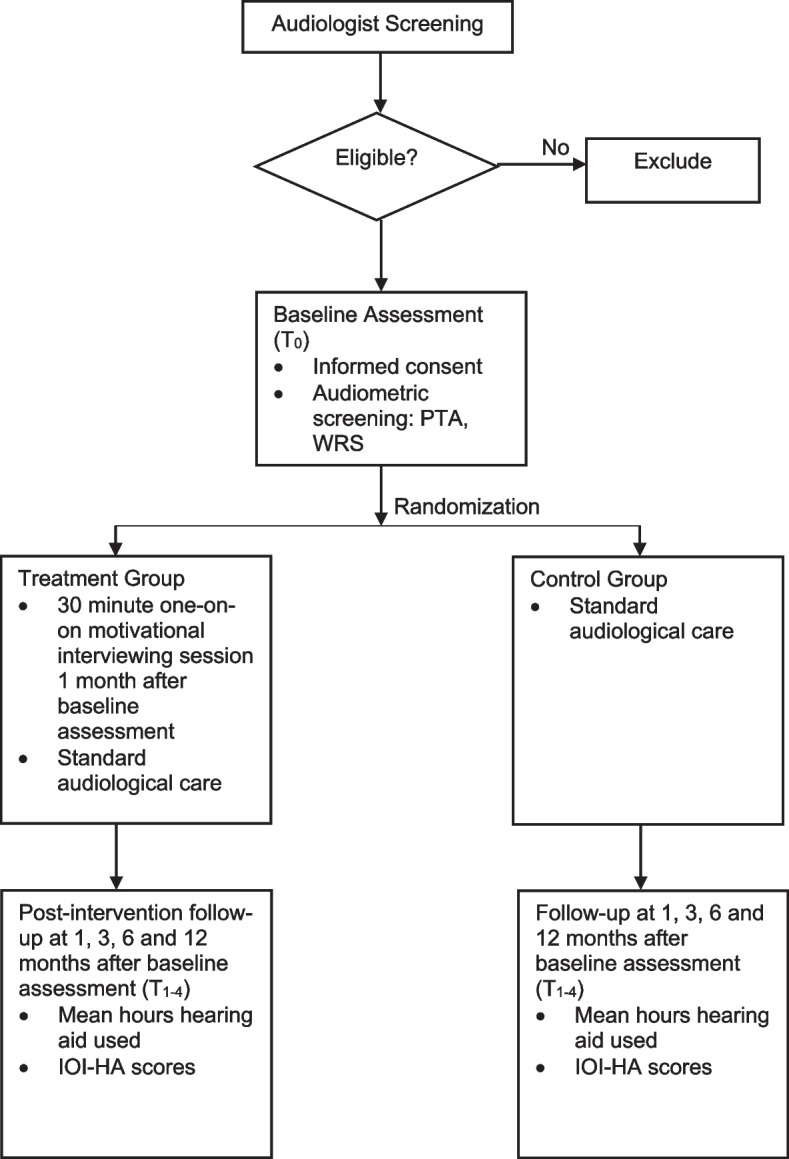
Participants’ screening and randomization

Figure 1 demonstrates the participant timeline for this study. Participating audiologists will screen potential candidates in their normal day-to-day work. Signed consent and baseline assessment will be obtained from those who meet inclusion criteria and agree to participate in the study. All participants will have a 1-month follow-up after the initial assessment with an audiologist in-person or via telehealth where IOI-HA questionnaires will be administered, and HA data-logging will be accessed. Those in the intervention group will also undergo a 30-min one-on-one MI session after the 1-month follow-up visit and the details will be provided by the study coordinator. Subsequent follow-up visits will be conducted at 3, 6, and 12 months where participants will complete the IOI-HA questionnaire and their HA data-logging will be accessed.

### Sample size {14}

Aazh et al.’s [6] feasibility investigation of assessing HA data-logged hours of use in response to MI concluded that a standardized difference of 0.6 was best for sample size estimation. Therefore to discern this difference or greater at a 5% significance level with a power of 80%, 45 patients will be required in each intervention arm based on Machin and Campbell’s [21] clinical trial design tables. Therefore, 90 participants in total at a randomization ratio of 1:1.

An allowance of 50% participant dropout rate was adopted in this study as opposed to the 20% drop out identified by Aazh et al. to compensate for the uncertainties or anticipated higher drop rate due to the COVID-19 pandemic [6]. This higher rate is reasonable because a previous hearing aid RCT trial conducted by our senior investigators who recruited participants from audiology clinics within the greater Vancouver area, in part over the pandemic period with 3 months’ follow-up, resulted in 39.1% of participant data in the intervention arm being unavailable because of participants’ failure to attend follow-up or complete study questionnaires fully [24]. This leads to an adjusted sample size of 180 participants, 90 per group.

### Recruitment {15}

The estimated time period for recruitment, intervention, and data acquisition is 48 months. Investigating and collaborating with audiologists will invite eligible clients attending their clinics between January 2021 and December 2024 to participate in the study. The time requirement for participant recruitment and baseline assessment will be approximately 60 to 75 min.

## Assignment of interventions: allocation

### Sequence generation {16a}

Participants will be allocated to the study group by block randomization. The block sequence will be generated using the computer-generated STATA statistical package (Version 16). Block size variation will be used to prevent the prediction of treatment. Allocationwill be performed by the study coordinator who will not be involved in data collection; patients will be assigned a non-identifiable number sequence.

### Concealment mechanism {16b}

As stated above, participants will be allocated to treatment or control groups using block randomization after being recruited by audiology clinics. Allocation will be done by the study coordinator in a sequentially numbered fashion using opaque, sealed envelopes. The study coordinator will only know one patient outcome of intervention at a time to inform the MI therapist and patient. The recruiting audiologists will be blinded to study group allocation and do not have access to the allocation sequence. Only the MI therapist and study coordinator will be informed of patient group allocation after recruitment.

### Implementation {16c}

Participating audiologists and the trained clinic staff will enroll eligible participants. Assignment to the intervention and control groups will be done independently by the study coordinator.

## Assignment of interventions: blinding

### Who will be blinded {17a}

Audiologists and the investigators at the time of outcome data collection will not be blinded to study group allocation. The analysis team will receive aggregated data obtained for control and intervention groups. Study participants will become aware of their group allocation as the study progresses, but they will be asked not to reveal their group allocation to the treating audiologist or other investigators.

### Procedure for unblinding if needed {17b}

In the unlikely event where a participant encounters a serious adverse event, the principal investigator (PI) and the study coordinator will unlock that participant’s allocation code to investigate the event.

## Data collection and management

### Plans for assessment and collection of outcomes {18a}

Consented participants will be instructed about the study and its procedures. At the initial visit, data collection forms, including the email addresses of each participant, will be filled and collected by a trained audiology clinic assistant. This data will be securely stored on a password-protected device and transferred to the study coordinator and PI for long-term use and protection.

Outcome assessments of data-logged hearing hours and IOI-HA questionnaires will be performed at 1-, 3-, 6-, and 12-month time points after hearing aid fitting.

#### Pre-intervention assessment at 1 month

Participants in both groups will continue to be contacted by their audiology clinics as required for standard care at the above-mentioned time point. Participants in the MI intervention group will attend a virtual one-on-one counseling session after their 1-month follow-up from hearing aid fitting. Teleconferencing software will be used to organize this meeting and instructions will be provided via email to each participant or a family member by study coordinator. The total time is anticipated to be approximately 30 min. This session will be hosted by a practicing MI therapist who will explore and elicit motivation in the participants to use their HAs for more hours.

#### Post-intervention assessment at 3-, 6-, and 12-month follow-ups

Post interventions will be performed at 3-, 6-, and 12-month follow-ups from hearing aid fitting. During these follow-ups, in addition to standard care, participants will have the number of data-logged hearing hours per day extracted from their hearing device automatic data-logging feature and will be asked to complete the online IOI-HA questionnaires. The IOI-HA has been previously tested for its validity, reliability, and sensitivity [22, 23]. There have been suggestions that item 5 on the IOI-HA contributes inconsistently [22, 23]. However, the introspective subscale (items 1, 2, 4, and 7) of the IOI-HA was found to have a Cronbach’s alpha of 0.85 [23]. It has previously been used in other hearing aid outcome research [6].

### Plans to promote participant retention and complete follow-up {18b}

A member from the audiology clinic will complete all forms along with participants to ensure the completeness of the forms. These standardized forms have been revised to improve understandability to ensure completeness. In the event of withdrawal prior to completion, any data collected up to the withdrawal time point will be retained and included for data analyses. A SPIRIT flow diagram demonstrates the data collection in the intervention and control groups (Table 2) [25].

**Table 2:** Schedule of enrollment, interventions, and assessments

**Timepoint**	**Study period**											
	**Enrollment**		**Allocation**		**Post-allocation**				**Close-out**			
	***−t***_***1***_ −48 months to −1 day		***t***_***0***_ Day 0		***t***_***1***_ 1 month		***t***_***2***_ 3 months		***t***_***3***_ 6 months		***t***_***4***_ 12 months	
	**T**^**a**^	**C**^**b**^	**T**^**a**^	**C**^**b**^	**T**^**a**^	**C**^**b**^	**T**^**a**^	**C**^**b**^	**T**^**a**^	**C**^**b**^	**T**^**a**^	**C**^**b**^
**Enrollment:**												
Eligibility screen	X	X										
Informed consent	X	X										
Allocation			X	X								
**Intervention:**												
One-on-one MI					X							
Standard care						X	X	X	X	X	X	X
**Assessments:**												
Baseline assessment												
PTA			X	X								
WRS			X	X								
Primary outcomes												
Mean hours hearing aid used					X	X	X	X	X	X	X	X
IOI-HA score					X	X	X	X	X	X	X	X

*T* Treatment group, *C* Control group, *MI* Motivational interviewing, *PTA* Pure tone audiometry, *WRS* Word recognition score, *IOI-HA* International Outcome Inventory for Hearing Aids

### Data management {19}

To minimize data-entry errors, the data entry will be double-checked for errors or omissions by a study investigator blinded to group allocations. All consent forms and data will be managed by the study coordinator for secure documentation. Data will be stored in password-protected encrypted computers and physically held in patient charts.

### Confidentiality {27}

Participants will have the opportunity to have an informed discussion with study investigators regarding the trial when being screened for eligibility and prior to being consented. Participant will be assigned to a unique identification number to maintain the patient confidentiality.

### Plans for collection, laboratory evaluation, and storage of biological specimens for genetic or molecular analysis in this trial/future use {33}

N/a. There are no biological specimens that will be collected in this study.

## Statistical methods

### Statistical methods for primary and secondary outcomes {20a}

Descriptive Statistics will be used to summarize patient demographics. Independent samples *T*-tests will be used to compare continuous variables such as PTA averages, word recognition scores, and data-logged hearing hours between groups. Chi-squared test will be used to determine the degree of association between categorical variables such as patient-reported outcome, sex, and age group. A univariate linear regression will be run with demographic variables and data-logged hearing hours. Pearson’s correlations will also be used to measure the strength of associations between data-logged hearing aid use hours and self-reported measures via the IOI-HA questionnaire. Data will be analyzed using SPSS (Version 25).

### Interim analyses {21b}

Initial interim analysis will be performed when 30% of the participant recruitment is achieved to determine that the intake characteristics (age, sex, and degree of hearing loss) are similar in both arms. A second interim analysis will be performed when 50% of the recruited participants’ follow-up data is available to determine if a difference is evident in the outcomes. The results will guide the decision to continue the study.

### Methods for additional analyses (e.g., subgroup analyses) {20b}

There are no planned additional subgroup analyses.

### Methods in analysis to handle protocol non-adherence and any statistical methods to handle missing data {20c}

Sample population to be analyzed will be based on randomization. Any participant’s missing age data will be imputed with the mean of age of their allocation group. Missing sex data will not be analyzed. Participants without or with incomplete main outcome data including data-logged HA use hours and IOI-HA scores will be excluded from analysis.

### Plans to give access to the full protocol, participant-level data, and statistical code {31c}

The full protocol will be available for public access. Participant-level dataset and statistical code will not be available for public access.

## Oversight and monitoring

### Composition of the coordinating center and trial steering committee {5d}

The coordinating center and trial steering group (TSC) will consist of PI, study co-ordinator, and a research assistant.

### Composition of the data monitoring committee, its role, and reporting structure {21a}

A data monitoring committee is not needed over the course of this study as there is a low risk of adverse events.

### Adverse event reporting and harms {22}

After participants have provided consent and enrolled in the study, adverse events will be collected and recorded until the end of the study period. Adverse events that occur after consent is signed, but before receiving the study intervention will be reported as not related to our intervention. Any serious adverse event that occurs will be reported to the institutional review board (IRB) and will be documented.

### Frequency and plans for auditing trial conduct {23}

Study-related monitoring, audits, and inspections by the clinical research ethics board of all study-related documents will be performed when 30% of participant recruitment is achieved. The PI will also ensure the legitimacy and capability for inspections of facilities. A second audit will be performed when 50% of the recruited participants’ follow-up data is available. The results will guide the decision to continue the study.

### Plans for communicating important protocol amendments to relevant parties (e.g., trial participants, ethical committees) {25}

Any modifications to the protocol including changes in study objectives, study design, participants, sample size, or study procedures will require a formal amendment to the protocol and approval by the IRB prior to implementation.

### Dissemination plans {31a}

The PI will have access to the data sets. The results of this trial will be disseminated to the public through peer-reviewed research publications, conference abstracts, meeting presentations, posters, and social media. The study will report results based on the Consolidated Standards of Reporting Trials guidelines.

## Discussion

Hearing loss is the third leading cause of human disability and has negative effects on quality of life. HAs can improve one’s quality of life; however, there are many barriers to HA use [2]. There is conflicting evidence for MI and its effect on increasing HA use among new adult HA clients. This paper describes a protocol for a comprehensive randomized controlled trial to evaluate the impact of one-on-one MI counseling session on HA use in new adult HA clients.

The strengths of this study include the robust randomized controlled trial design, use of validated outcome measures, and longer-term follow-ups than in previous studies. The protocol of this study was developed with multidisciplinary input to ensure that it is robust, practicable, and applicable to local HAs dispensing practices. The multidisciplinary team included a practicing physician/surgeon who manages hearing loss and is experienced in randomized clinical trials, a psychologist with an extensive knowledge of MI, an audiologist experienced in dispensing HAs to the local population, and other study investigators with experience in conducting and analyzing research findings. While our study is being conducted in Vancouver, Canada, the protocol and procedures can be replicated in other regions and countries. This study will contribute to building the evidence around the effectiveness of one-on-one MI counseling on HA use.

The trial limitations are those that are shared with other RCT-designed studies. It is impossible to blind participants to their receipt of the intervention, a one-on-one interview with a MI therapist. However, masking will be maintained from study enrollment, group assignment, and up to the point where the participants are informed of their MI session appointment. Group allocation lists will be done by the study coordinator, who will not be involved in data collection to reduce bias. In addition, participants in the control group might be induced by questions in the IOI-HA questionnaire to alter their behavior. Therefore, it is possible that induced change in the control group may reduce the ability to find differences in the outcomes between the intervention and control groups. Lastly, this study is limited by the realities of HA funding in the current healthcare setting, which limits participant’s choice and other similar biases.

To conclude, whether one-on-one MI can improve HA adherence among new adult clients is lacking compelling evidence, but it could have substantial clinical, social, and public health impacts for people with hearing loss. When completed, our study should provide further evidence on the effect of one-on-on MI in HA outcomes in adults with hearing loss.

## Trial status

The current protocol is version 9.0, dated August 17, 2022. Recruitment of patients began in March 2021 and is expected to end in December 2023. The data collection will run until all participants complete their follow-ups. The study is expected to run until the end of December 2024. Until then, the intervention effects are unknown.

## Supplementary Information

**Additional file 1.** Consent form.

## Data Availability

The principal investigator and study coordinator will have access to the final trial dataset. Any data required to support the protocol can be supplied on request.

## Abbreviations

HA: Hearing aid
HRQoL: Health-related quality of life
MI: Motivational interviewing
IOI-HA: International Outcome Inventory for Hearing Aids
PTA: Pure tone audiometry
