# Supplementary material for: A randomized controlled trial evaluating the effects of motivational interviewing in new hearing aid users (MI-HAT): study protocol for a randomized controlled trial
Source: Trials. 2023 May 22;24:346. doi: 10.1186/s13063-023-07352-7 (PMC10201034; doi:10.1186/s13063-023-07352-7)
Supplement: Supplementary file 1 — Additional file 1. Consent form. [file 13063_2023_7352_MOESM1_ESM.docx]

**RESEARCH PARTICIPANT INFORMED CONSENT FORM**

| **1. Title** | **A Randomized Controlled Trial Evaluating the Effects of Motivational Interviewing in New Hearing Aid Users (MI-HAT)** |
| --- | --- |
| **2. Study Personnel** |  |
| **Principal Investigator** | Dr. Desmond Nunez *MD, MBA, FRCS(ORL), FRCSC*  Div. of Otolaryngology, Dept. of Surgery,  The Uni. of British Columbia  Gordon & Leslie Diamond Health Care Centre  4th. Fl. 2775 Laurel Street,  Vancouver General Hospital,  Vancouver, BC, V5Z 1M9  Tel: (+1) 604-875-4664 |
| **Primary Contact** | [Reyhaneh Abgoon](https://rise.ubc.ca/rise/sd/Rooms/DisplayPages/LayoutInitial?Container=com.webridge.entity.Entity%5bOID%5b444546701806C540B47F7DC3E5F1EB57%5d%5d)  Div. of Otolaryngology, Dept. of Surgery,  The Uni. of British Columbia  Gordon & Leslie Diamond Health Care Centre  Vancouver, BC, V5Z 1M9  Email: reyhaneh.abgoon@ubc.ca |
| **Sponsor(s) / Funder** | None |

**3. Invitation**

You are invited to take a part in this research study because you are 18 years and older, have not used a hearing aid before, and have a hearing level that could be improved with a hearing aid.

**4. Your participation is voluntary**

Your participation is voluntary. You have the right to refuse to participate in this study. If you decide to participate, you may still choose to withdraw from the study at any time without any negative consequences to the medical care, education, or other services to which you are entitled or are presently receiving.

Please review the consent document carefully when deciding whether or not you wish to be part of the research and sign this consent only if you accept being a research participant.

**5. Who is conducting the study?**

This study is not receiving funds from an external agency or sponsor. This study will be conducted by Dr. Desmond A. Nunez, Dr. Alice Liu, Ms. Carol Lau, Dr. Jane Sun, Ms. Joyce Liu and Ms. Shirley Young.

**6. Background**

Hearing loss is one of the most common chronic disabilities in the older adult population. The world health organization reports that 33% of the global population aged over 65 years have disabling hearing loss. In Canada, 65% of Canadians who are 70 and older suffer from hearing loss in frequencies associated with normal speech. Those with hearing loss are more likely to have a lower quality of life. They may experience anxiety, depression, safety concerns, lack of mobility, and reduced employment opportunities and income. Hearing aid use can improve one’s quality of life by increasing a person’s ability to detect, differentiate and locate sound, improve speech recognition.

Several factors seem to reduce motivation to use a hearing aid. Fears of exclusion and shame due to hearing loss are major deterrents to hearing aid use. It is necessary for hearing aid fitting professionals to consider these user needs so that patients feel more comfortable using them. A popular suggestion in current research is to implement counselling sessions in the hearing aid fitting process.

**7. What is the purpose of the study?**

The purpose of this study is to determine if a one-on-one counselling may improve hearing aid use in addition to standard care. This study will also seek to determine if this approach has any ill-effects.

**8. Who can participate in this study?**

You may be able to participate in this study if*:*

- You have a hearing ability that could be improved with a hearing aid in one or both ears, as determined by your audiologist.

**9. Who should not participate in the study?**

You will not be eligible to participate in this study if:

- You are unable to complete the questionnaires in English
- Your audiologist determines that your hearing tests responses are variable.

**10. What does the study involve?**

If you agree to take part in this study, the specific procedures and visits you can expect will include the following:

- **Participant recruitment**

The initial study portion will take approximately 10-15 minutes to complete the consent form by an interested participant and a data collection form by a trained audiology clinic assistant at the audiology clinics. Your hearing aid will be tracked to determine the hours of use. Afterwards, you will be randomly assigned to either a group with counselling session and standard care, or a group with standard care only. You will then be offered suitable dates & times for your study visit in one month.

- **Study visits**

There are 4 expected study visits.

1. At 1-month (Pre-treatment)

You will be asked to fill out an online questionnaire regarding your hearing aid use. The average number of hours the hearing aid is used will be collected by the audiology clinic. A total of 10-15 minutes will be required to complete this assessment.

At this time point, if you are randomized to treatment group, you will have a one-on-one counselling session via Zoom teleconference in addition to standard care. This session will last a maximum of 30 minutes. Your care provider may ask questions related to hearing aid use.

If you are randomized to control group, you will receive only standard care from your audiology clinic.

1. At 3, 6, and 12-month follow-ups (Post-treatment)

During the 3, 6, and 12 month follow-ups, you will be offered standard care treatments such as education, hearing aid adjustments, and practice using hearing aid at the audiology clinic. Average number of hours of hearing aid use will be collected by the audiology clinics. You will be also asked to fill out an online questionnaire about your hearing aid use at each follow-up. This will take approximately 10-15 minutes apart from standard care.

**11. What are the possible harms and discomforts?**

There are no known risks for this study. You do not have to answer any questions that you are uncomfortable answering.

**12. What are the potential benefits of participating?**

There may be no direct benefit to you from taking part in this study. However, your hearing aid use may increase, leading to a greater improvement in your quality of life. We hope that the information learned from this study can be used in the future to benefit other hearing aid users.

**13. After the study is finished**

If the study determines that one-on-one motivational interviewing counseling helps to increase hearing aid use, then any participants who have not received motivational interviewing during the study will be offered the treatment.

**14. What happens if I decide to withdraw my consent to participate?**

You may withdraw from this study at any time without giving reasons. If you choose to enter the study and then decide to withdraw at a later time, you have the right to request the withdrawal of your information collected during the study. This request will be respected to the extent possible. Please note however that there may be exceptions where the data will not be able to be withdrawn, for example where the data is no longer identifiable (meaning it cannot be linked in any way back to your identity) or where the data has been merged with other data. If you would like to request the withdrawal of your data, please let the Principal Investigator of the study know.

**15. How will my taking part in this study be kept confidential?**

Your confidentiality will be respected. However, research records and health or other source records identifying you may be inspected in the presence of the Investigator or designate and by representatives of UBC’s Clinical Research Ethics Board, for the purpose of monitoring the research. No information or records that disclose your identity will be published without your consent, nor will any information or records that disclose your identity be removed or released without your consent unless required by law. Research findings will be published in a prestigious journal and it will be distributed via email by study coordinator to research participants if requested.

You will be assigned a unique study number as a participant in this study. This number will not include any personal information that could identify you (e.g., it will not include your Personal Health Number, SIN, or your initials, etc.). Only this number will be used on any research-related information collected about you during the course of this study, so that your identity will be kept confidential. Information that contains your identity will remain only with the Principle Investigator and/or designate. The list that matches your name to the unique study number that is used on your research-related information will not be removed or released without your consent unless required by law.

Your rights to privacy are legally protected by federal and provincial laws that require safeguards to insure that your privacy is respected. You also have the legal right of access to the information about you and, if need be, an opportunity to correct any errors in this information. Further details about these laws are available on request to your study team.

**Disclosures associated with Zoom conference**

The Zoom conferencing will be organized using UBC licensed Zoom. In addition, you are committing to confidentiality measures: not to share meeting invites or information links on social media or public events, not invite or allow others to attend the meeting with you, not record your conversation with your care providers and introduce yourself to care provider by name. You can protect your identity and increase the protection of your personal information by using only a nickname or a substitute name and by turn off your camera. We encourage all participants to refrain from disclosing the contents of the discussion outside of the counselling session; however, we cannot control what participants do with the information discussed.

If you wish to email your completed consent form back to researchers, you should know that this will likely go through servers outside of Canada and thus may increase the risk of disclosure because the laws in those countries, dealing with protection of information may not be as strict as in Canada.

**Disclosure of Race/Ethnicity**

Studies involving humans now routinely collect information on race and ethnic origin as well as other characteristics of individuals because these characteristics may influence how people respond to different medications. You should be aware that providing this information is not mandatory.

**16. What happens if something goes wrong?**

By signing this form, you do not give up any of your legal rights and you do not release the Principle Investigator, participating institutions, or anyone else from their legal and professional duties.

**17. What will the study cost me?**

All research-related procedures that you will receive during your participation in this study will be provided at no cost to you.

**Reimbursement**

You will not be reimbursed for any expenses incurred (i.e. parking, transportation) from participating in this study.

**Remuneration**

You will not be remunerated for participating in this study.

**18. If I have questions about the study procedures during my participation, who should I speak to?**

If you have any questions or desire further information about this study before or during participation, or if you experience any adverse effects, you can contact the Principal Investigator, Dr. Desmond A. Nunez at 604-875-4664.

**19. Who do I contact if I have any questions or concerns about my rights as a participant?**

If you have any concerns or complaints about your rights as a research participant and/or your experiences while participating in this study, contact the Research Participant Complaint Line in the University of British Columbia Office of Research Ethics by e-mail at RSIL@ors.ubc.ca or by phone at 604-822-8598 (Toll Free: 1-877-822-8598). Please reference the study number (H20-02393) when calling so the Complaint Line staff can better assist you.

**20. Signatures**

**Participant Consent**

My signature on this consent form means:

- I have read and understood the information in this consent form.
- I have been able to ask questions and have had satisfactory responses to my questions.
- I understand that my participation in this study is voluntary.
- I understand that I am completely free at any time to refuse to participate or to withdraw from this study at any time.
- I understand that I am not waiving any of my legal rights as a result of signing this consent form.
- I understand that there is no guarantee that this study will provide any benefits to me.
- I will receive a signed copy of this consent form for my own records.
- I consent to participate in this study.

| **Participant:** | | | | | | | | | | |
| --- | --- | --- | --- | --- | --- | --- | --- | --- | --- | --- |
|  | | |  |  | | |  | |  | |
| Name of Participant (Print) | | |  | Signature of Participant | | |  | | Date (yyyy-mm-dd) | |
|  | | | | | | | | | | |
| **Person obtaining informed consent:** | | | | | | | | | | |
|  |  |  | | |  |  | |  | |  |
| Name (Print) |  | Signature | | |  | Study Role | |  | | Date(yyyy-mm-dd) |
| **Future Contact**  Are you interested in learning about other studies conducted by Dr. Desmond A. Nunez in the future?  ⬜ Yes ⬜ No Initials___________  Note that for any future studies, a separate consent form will be provided to you for review. | | | | | | | | | | |
